# Role of LOX-1 and ROS in oxidized low-density lipoprotein induced epithelial-mesenchymal transition of NRK52E

**DOI:** 10.1186/1476-511X-9-120

**Published:** 2010-10-19

**Authors:** Rui Wang, Guohua Ding, Wei Liang, Cheng Chen, Hongxia Yang

**Affiliations:** 1 Division of Nephrology, Renmin Hospital of Wuhan University, Wuhan 430060, China

## Abstract

**Background:**

To investigate the effect of oxidized low density lipoprotein receptor-1 (LOX-1) on tubular epithelial-mesenchymal transition (TEMT) induced by oxidized low-density lipoprotein (ox-LDL) and its mechanism.

**Methods:**

NRK-52E cells were incubated with ox-LDL (0, 25, 50, and 100 μg/ml) for 24 hours or pre-treated with the chemical inhibitor of the LOX-1 receptor polyinosinic acid (poly I) and carrageenan or the antioxidant N-acetyl-L-cysteine (NAC), the cells were then exposed to 50 μg/ml of ox-LDL.The expression of LOX-I, E-cadherin, α-smooth muscle actin (α-SMA) and reactive oxygen species (ROS) were analyzed by real-time PCR, western blotting analysis, immunofluorescence and confocal laser scanning microscopy.

**Results:**

Ox-LDL increased the expression of LOX-1 mRNA and protein in a dose-dependent manner from 0 to 100 μg/ml (P < 0.05). Following the increase in the LOX-1 protein level, the lipid intake, ROS generation and α-SMA expression increased; however, the E-cadherin level decreased. The pre-treatment with poly I or carrageenan or NAC significantly inhibited the LOX-1 expression, α-SMA expression, the lipid intake and ROS generation and reversed decrease of E-cadherin expression induced by ox-LDL. Meanwhile, the ROS generation were associated with a increase in the LOX-1 expression. The α-SMA expression was positively correlated with the ROS generation and LOX-1 expression, and the E-cadherin expression was negatively correlated with the ROS generation and LOX-1 expression.

**Conclusions:**

LOX-1 and ROS may play a important role in epithelial-mesenchymal transition of NRK52E induced by OX-LDL.

## Introduction

Several studies have revealed that anoxia, ischemia and many cytokines can induce the tubular TEMT. Stephanie et al reported that a short-term diet of high cholesterol could induce a rise in oxidative stress and that ox-LDL could induce the activation of different intercellular cytokines among the interstitial tubular epithelial cells and alter the expression of certain genes, which leads to renal tubular interstitial fibrosis[1]. However, there are few reports regarding the TEMT induced by ox-LDL, and whether ROS is involved remains unknown. LOX-1 was recently found to be one of the ox-LDL receptors expressed in many cells. It is involved not only in the cellular interaction and adhesion, signal transduction and mediation of the digestion and degradation of ox-LDL but also in many pathological processes, including ischemia-reperfusion injured myocardium, atherosclerosis, rheumatoid arthritis (RA) and kidney injury of diabetic nephropathy. Currently, there is some controversy as to whether the LOX-1 is expressed in the tubular epithelial cells. This study investigates the effects of the lectin-like oxidized low density lipoprotein receptor-1 (LOX-1) in TEMT induced by ox-LDL and its mechanism.

## Materials and methods

### Main reagents

FBS and DMEM (Gibco, USA), NRK-52E (ATCC, USA), anti-rat LOX-1 goat antibody (Santa Cruz Biotechnology, USA), β-actin (Santa Cruz Biotechnology, USA), anti-rat mouse E-cadherin monoclonal antibody (BD, USA), and mouse α-SMA monoclonal antibody (Boster, P.R.China) were purchased from the corporations given. The N-acetylcysteine (NAC), Oil Red O, polyinosinic I (Poly I), carrageenan (Sigma, USA), the fluorescent probe CM-H__2__DCFDA (Molecular Probes, USA), and Trizol (Invitrogen, USA) were purchased from the corporations shown. A chemiluminescent kit (Santa Cruz Biotechnology, USA) was purchased, and the LOX-1 primers and fluorescent probes were synthesized by Da an Gene in China. The SuperScript™ First-Strand reaction synthesis system kit was purchased from Fermentas, USA, and the SYBR Green PCR Master Mix was purchased from ABI, USA.

### Preparation of oxidized low density lipoprotein (ox-LDL)

The native LDL (n-LDL) was prepared by a one-step density gradient ultracentrifugation and determined by the Lowry method. The n-LDL was incubated in a 5 μmol/L Cu^2+ ^solution for 24 hours at 37°C, and then placed in PBS with 200 μmol/ml EDTA at 4°C for 24 hours for dialysis. Subsequently, it was sterilized by a 0.45 μm micro-filter and stored at 4°C in the dark. The modification level was determined by the thiobarbituric acid (TBA) test.

### Cell culture of NRK-52E and grouping

NRK-52E were cultured in DMEM medium with 5% newborn calf serum, 100 IU/ml penicillin, 100 μg/ml streptomycin in 5% CO2 at 37°C,Cells were incubated for 24 hours with 0.5% newborn calf serum before ox-LDL stimulation.

Grouping for each experiment:

1. Group of different doses of ox-LDL: different concentrations of ox-LDL (0, 25, 50 and 100 μg/ml) were added into the medium for incubation for 24 hours;

2. Group of Lox-1 inhibitors: samples were pretreated with Poly I (250 μg/ml) or carrageenan (250 μg/ml) or NAC for incubation for 2 hours, and then ox-LDL was added at the final concentration of 50 μg/ml in the ox-LDL medium for 24 hours.

### Real-time PCR for the Detection of the LOX-1 mRNA Level

Trizol was applied to extract the total RNA, and reverse transcription was conducted in a 20 μl reaction system. Using an UNO II PCR machine, quantitative fluorescent PCR was performed under the following conditions:

The temperature was 95°C for 5 min, 94°C for 20 s, 57°C for 20 s, 72°C for 20 s, 72°C for 5 min, and 55°C for 10 s; 55 cycles were used. The 18S RNA was used for internal control. The primers were designed and synthesized as follows: primers LOX-1 forward: 5'-GCCTAGTGTTATCAGTGACC-3', anti-sense 3'-CTTAGTTTCTCCCTTGACTTC-5'; and 18S sense: 5'-CCGAGAAGTTTCAGCACATCC-3', anti-sense: 5'-TGGCAGTGATAGCGAAGGCT-3'.

### Western blotting for the detection of LOX-1, α-SMA and E-cadherin

The cells were lysed by a 4°C pretreated cell lysis solution, and the total protein level was determined by the Comas blue method. The samples with 30 μg total protein each were applied for SDS-PAGE and were electro-transferred to the NC membrane. In addition, 5% lipid-free milk powder was used for blocking for 2 hours. The anti-rat goat LOX-1 (1:200), α-SMA(1:100) and anti-E-cadherin antibody (1:600) were added separately, and the membrane was incubated at 4°C overnight. After washing, the anti-goat and anti-rabbit IgG antibody linked with HRP were added for incubation for 1 hour, and the exposed film was later developed. Using β-actin as the internal control, the relative protein expression levels were determined quantitatively using an imaging analyzing system to scan the absorbance of specific bands.

### Oil Red O Staining

The cell cover slides were washed with PBS three times for 5 minutes each and fixed with 50% isopropanol for 1 minute.Cell were stained 10 minutes with Oil Red O and then washed with distilled water. Hematoxylin was applied for 5 minutes for staining, and the images were collected by a PIAS-1000 imaging analysis system after color separation.

### Confocal Laser for the Detection of the Intracellular ROS Level

Intracellular ROS was labeled with CM-H__2__DCFDA, which is a fluorescent probe. The CM-H__2__DCFDA storage solution was diluted to 10 μM by PBS with 2% glucose before usage. The medium was discarded, and the cell cover slides were added with 0.5 ml CM-H__2__DCFDA for incubation for 45 minutes in the dark. Then confocal laser scanning was performed, and the results were analyzed by the Leica Confocal Software.

### Statistics

Data were analyzed by SPSS 11.0 software, and the one-way analysis of variance (ANOVA) was applied for inter-group comparison. The LSD test was applied for the one-to-one comparison, and the Pearson's test was applied for the correlation analysis. P < 0.05 was considered significant.

## Results

### The influence of ox-LDL, LOX-1 inhibitor and NAC on the LOX-1 mRNA and protein levels

**Figure 1:**
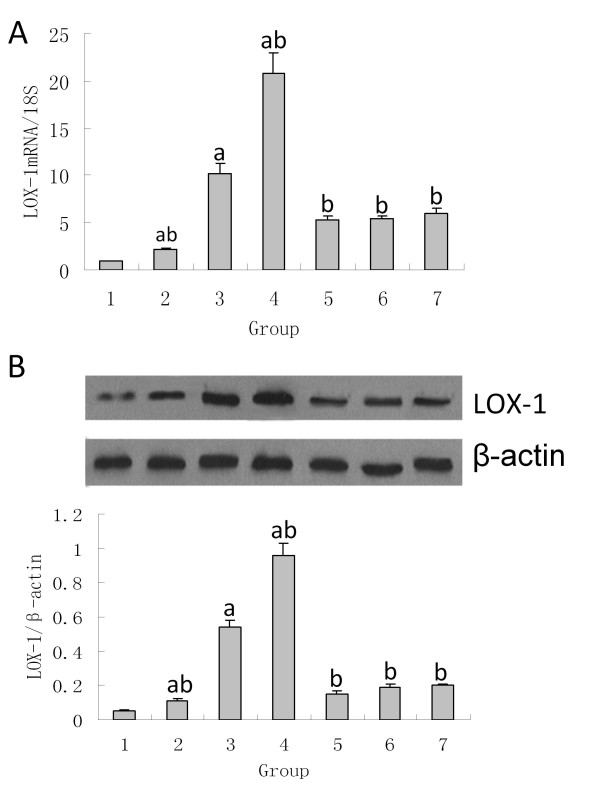
**The influence of ox-LDL, LOX-1 inhibitor and NAC on the LOX-1 mRNA and protein levels.**  A: The expression of LOX-1mRNA. Within the range of 0-100 μg/ml, the ox-LDL enhances the LOX-1 mRNA transcription in NRK52E cells in a dose-dependent manner. The LOX-1 inhibitor poly I and carrageenan and the antioxidant NAC can suppress the LOX-1 mRNA transcription induced by ox-LDL in NRK52E cells. B: The expression of the LOX-1 protein. Within the range of 0-100 μg/ml ox-LDL enhances the LOX-1 protein expression in NRK52E cells in a dose-dependent manner. The LOX-1 inhibitor poly I and carrageenan and the antioxidant NAC can suppress the LOX-1 protein expression induced by ox-LDL in NRK52E cells. Note: 1: 0 μg/ml ox-LDL; 2: 25 μg/ml ox-LDL; 3: 50 μg/ml ox-LDL; 4: 100 μg/ml ox-LDL; 5: poly I + 50 μg/ml ox-LDL; 6: carrageenan + 50 μg/ml ox-LDL; 7: NAC + 50 μg/ml ox-LDL; comparison with 0 μg/ml ox-LDL, ^a^*P *< 0.05; comparison with 50 μg/ml ox-LDL, ^b^*P *< 0.05.

Low levels of LOX-1 mRNA and protein are expressed in normal NRK52E cells. In this study, the different concentrations of ox-LDL significantly enhanced the expression levels of LOX-1 mRNA and protein in a dose-dependent manner, with concentrations ranging from 0 to 100 μg/ml, in comparison with the controls. The samples pretreated with poly I, carrageenan or NAC showed significant repression of the upregulated LOX-1 mRNA and protein levels induced by the ox-LDL (Figures 1).

### The influence of Ox-LDL, LOX-1 inhibitor and NAC stimulant on the intracellular Lipids

In normal NRK52E cells, a few lipid drops stained with oil red O could be observed, and with the treatment of incremental concentrations of ox-LDL, the intracellular red oil O stained lipids increased. Meanwhile, the samples pretreated with poly I, carrageenan or NAC significantly reduced the red oil O stained lipids upon upregulation by the ox-LDL.

### The influence of Ox-LDL, LOX-1 inhibitor or NAC on the intracellular ROS

**Figure 2:**
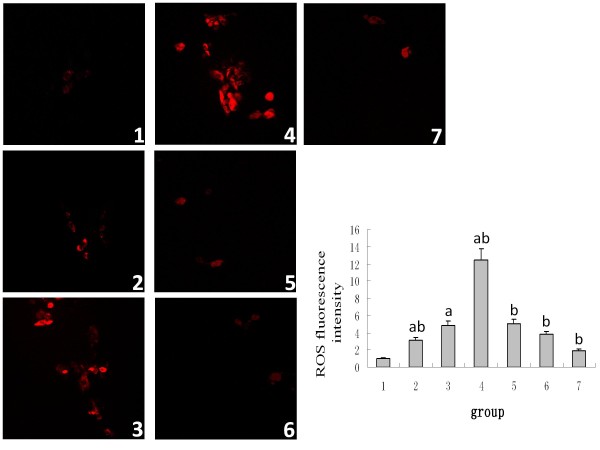
**The generation of intracellular ROS**. Within the range of 0-100 μg/ml, the ox-LDL induces the ROS generation in NRK52E cells, while the LOX-1 inhibitors including poly I and carrageenan and the antioxidant NAC prohibit the ROS generation induced by ox-LDL in NRK52E cells. Note: 1: 0 μg/ml ox-LDL; 2: 25 μg/ml ox-LDL; 3: 50 μg/ml ox-LDL; 4: 100 μg/ml ox-LDL; 5: poly I + 50 μg/ml ox-LDL; 6: carrageenan + 50 μg/ml ox-LDL; 7: NAC + 50 μg/ml ox-LDL; compared with the 0 μg/ml ox-LDL group, ^a^*P *< 0.05; compared with the 50 μg/ml ox-LDL group, ^b^*P *< 0.05.

No ROS were observed in the normal NRK52E cells; with the treatment of incremental concentrations of the ox-LDL stimulant, the expressions of LOX-1 and intracellular ROS increased. The samples treated with poly I or carrageenan revealed suppressed LOX-1 mRNA and protein expression induced by the ox-LDL; as a result, the intracellular ROS was reduced. When the NAC was applied to suppress the ROS generation, the LOX-1 expression was reduced accordingly. This suggests that the generation of ROS has a positive correlation with the LOX-1 expression, and the correlation coefficient (r) was found to be 0.91 (Figures 2).

### The influence of Ox-LDL, LOX-1 Inhibitor and NAC on NRK52E Cell Transdifferentiation

**Figure 3:**
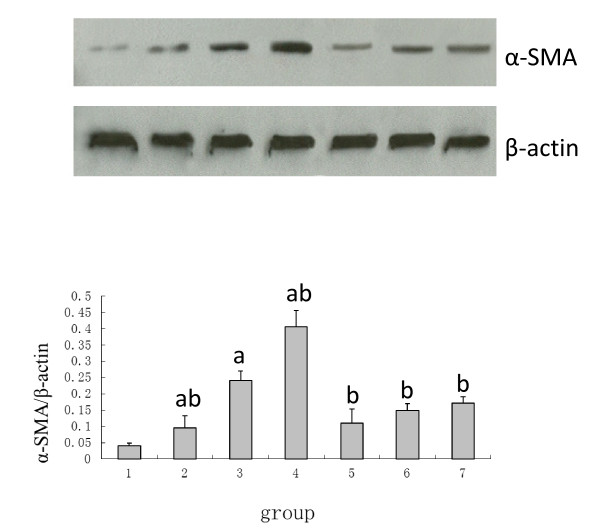
**The expression of α-SMA**. Within the range of 0-100 μg/ml, the ox-LDL stimulates the α-SMA expression in NRK52E cells in a dose-dependent manner, while the LOX-1 inhibitors including poly I and carrageenan and the antioxidant NAC suppress the expression of α-SMA induced by ox-LDL in NRK52E cells. Note: 1: 0 μg/ml ox-LDL; 2: 25 μg/ml ox-LDL; 3: 50 μg/ml ox-LDL; 4: 100 μg/ml ox-LDL; 5: poly I + 50 μg/ml ox-LDL; 6: carrageenan + 50 μg/ml ox-LDL; 7: NAC + 50 μg/ml ox-LDL.

**Figure 4:**
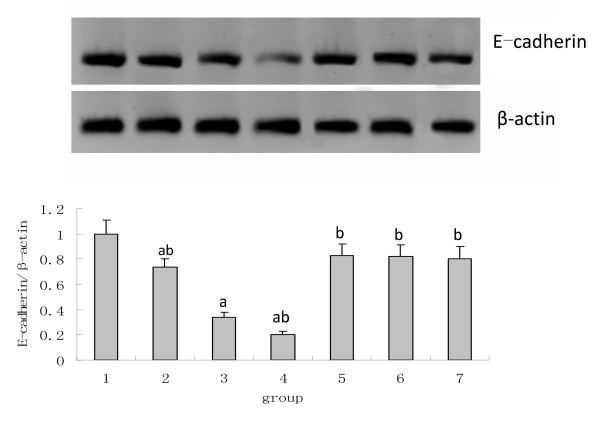
**Changes in the E-cadherin expression**. Within the range of 0-100 μg/ml, the ox-LDL decrease the expression of E-cadherin in tubular epithelial cells in a dose-dependent manner, while the LOX-1 inhibitors including poly I and carrageenan and the antioxidant NAC reverse the decrease of E-cadherin expression induced by ox-LDL in NRK52E cells. Note: 1: 0 μg/ml ox-LDL; 2: 25 μg/ml ox-LDL; 3: 50 μg/ml ox-LDL; 4: 100 μg/ml ox-LDL; 5: poly I + 50 μg/ml ox-LDL; 6: carrageenan + 50 μg/ml ox-LDL; 7: NAC + 50 μg/ml ox-LDL; compared with the 0 μg/ml ox-LDL group, ^a^*P *< 0.05; compared with the 50 μg/ml ox-LDL group, ^b^*P *< 0.05.

Little α-SMA expression was observed in the normal NRK52K cells; however, high levels of E-cadherin protein were observed. With the incremental LOX-1 expression and ROS, the α-SMA was increasingly expressed and was accompanied with a reduction of E-cadherin, the cells began transdifferentiation. The samples pretreated with poly I or carrageenan to block the LOX-1 or with NAC to suppress the ROS exhibited reduced α-SMA expression but increased E-cadherin expression. The α-SMA exhibited a close positive correlation with the LOX-1 and ROS, and the correlation coefficients were found to be 0.97 and 0.87, respectively. Meanwhile, the E-cadherin exhibited a close negative correlation with the LOX-1 and ROS, and the correlation coefficients were found to be -0.94 and -0.82, respectively (see Figures 3 and 4).

## Discussion

It is well-recognized that dyslipidemia can cause or worsen renal injuries including glomerulus, tubule and tubule-interstitial injuries, which display with tubular epithelial cell hypertrophy, inflammatory cell infiltration, cytokine generation and tubular interstitial fibrosis [2,3],but the mechanism is unclear.

LOX-1 is a recently discovered specific ox-LDL receptor that is found to be expressed in many kinds of cells to mediate multiple pathological injuries,such as ischemia-reperfusion,injured myocardium, atherosclerosis, rheumatoid arthritis. Applying an anti-LOX-1 treatment can reverse those pathological injuries. It is possible that LOX-1 could be a new target for the treatment of multiple diseases [4-8]. A few studies on LOX-1 expression in the kidney have suggested that LOX-1 might have an effect on the development of chronic renal disease by the evidence that anti-LOX-1 treatment can reverse enlarged kidney, oxidative stress, and leukocyte infiltration; can lower the level and function of mitochondria enzymes caused by diabetes; and can protect the renal microvascular bed [9-13]. However, it is still under debate whether LOX-1 is expressed in the tubular epithelial cells. By applying real-time PCR and western blot, our study showed that there is LOX-1 mRNA transcription and protein expression in normal NRK52E cells, confirming the reports by Jesus et al [10]. Furthermore, by using different concentrations of ox-LDL to stimulate the NRK52E cells, our study reveals that ox-LDL can up-regulate the mRNA and protein levels of LOX-1, and within the range of 0 to 100 μg/ml, the ox-LDL stimulates the LOX-1 expression in a dose-dependent manner.

Tubular EMT is the major mechanism in the development of tubular interstitial fibrosis, and it exhibits a loss of tubular polarity, the expression of α-SMA and vimentin (markers of mesenchymal cells) and the loss of E-cadherin and cytokeratin (markers of epithelial cells). Our study revealed that the ox-LDL stimulates the expression of α-SMA and the reduction of E-cadherin. Within the range from 0 up to 100 μg/ml, the increasing concentration of ox-LDL leads to higher α-SMA expression and lower levels of E-cadherin, suggesting that the ox-LDL enhances the TEMT of NRK52E in a dose-dependent manner. The cells pretreated with the LOX-1 inhibitor poly I and carrageenan, which suppressed the expression of the LOX-1, exhibited a reduction in the α-SMA expression and an increase in the E-cadherin expression. This suggested that the α-SMA has a positive correlation with LOX-1 and that E-cadherin has a negative correlation with LOX-1. Our study confirms that the ox-LDL induces the tubular TEMT through LOX-1.

ROS is usually considered to be a toxic by-product of cell metabolism. It interacts with lipids, proteins or DNA, leading to histological changes and cellular malfunction; furthermore, this reaction is different in different cells. During the process of renal fibrosis, ROS plays an important role in the synthesis and degradation of the extracellular matrix of the glomerulus and tubular interstitial tissue. It has also been reported that ROS is involved, through the activation of the MAPK/ERK pathway, in the tubular TEMT induced by TGF-β and aldosterone [14-16]. However, there are still no reports on whether the ox-LDL induces TEMT through ROS. Our study shows that the ox-LDL enhances the lipid intake and ROS generation, and the lipid intake reduces with the reduction of ROS after the inhibition of the LOX-1 expression by poly I and carrageenan. This suggests that the ox-LDL binds the LOX-1 to induce lipid intake, thus leading to the ROS generation. Meanwhile, after pretreatment with the antioxidant NAC and incubation with the ox-LDL, the cells exhibit partially reversed α-SMA expression up-regulated by the ox-LDL and E-cadherin expression down-regulated by the ox-LDL, thus suggesting ROS is one of the mechanisms of TEMT of NRK52E induced by ox-LDL. In addition, the expression of LOX-1 was impaired after the inhibition of ROS by NAC; this suggests that the ROS is not only the product of a combination of ox-LDL and LOX-1 but also the stimulant of the LOX-1 expression, where they act as positive feedback for each other.

In summary, ox-LDL enhances the LOX-1 expression in tubular epithelial cells in a dose-dependent manner within a certain concentration range. Ox-LDL binds to LOX-1 to mediate lipids into the cells to induce the ROS generation and TEMT of NRK52E through ROS. In addition, as a positive feedback, the ROS upregulates the LOX-1 expression and is thus involved in the TEMT of NRK52E. The suppression of LOX-1 can inhibit the ROS generation and TEMT of NRK52E to protect the normal tubular morphology.

## Competing interests

The authors declare that they have no competing interests.

## Authors' contributions

Conceived and designed the experiments: GD and RW. Performed the experiments: RW, WL and HY. Analyzed the data: RW, and WL. Wrote the paper: RW and CC. All authors read and approved the final manuscript.

## Acknowledgements

**Funding:** This work was supported by the National Natural Science Foundation  of China (30670895).
